# Fine needle aspiration biopsy diagnosis of dedifferentiated liposarcoma: Cytomorphology and MDM2 amplification by FISH

**DOI:** 10.4103/1742-6413.62257

**Published:** 2010-04-06

**Authors:** Hatem Q. Al-Maghraby, Walid E. Khalbuss, Uma N. M. Rao, Kathleen Cieply, Sanja Dacic, Sara E. Monaco

**Affiliations:** Department of Pathobiology and Laboratory Medicine, University of Toronto, Toronto, Ontario, Canada; 1 Department of Pathology, University of Pittsburgh Medical Center, Pittsburgh, Pennsylvania, USA

**Keywords:** Cytopathology, fine needle aspiration biopsy liposarcoma, *MDM2*

## Abstract

Lipomatous mesenchymal tumors constitute the most common type of soft tissue tumors. Well-differentiated liposarcoma (WDLS) can undergo dedifferentiation to a nonlipogenic sarcoma of variable histologic grade. In the recent literature, amplification of the murine double minute 2 *(MDM2)* oncogene, which has a role in cell cycle control, has been successful in distinguishing WDLS from benign lesions. We present a case of dedifferentiated liposarcoma diagnosed by fine-needle aspiration (FNA), using cytomorphology and ancillary studies (immunocytochemistry and fluorescent in-situ hybridization). An 85-year old female presented to our institution with a firm soft tissue mass of the right buttock. The FNA showed atypical spindle cells, osteoclast-like giant cells and extracellular dense matrix material. The cell block showed cellular groups of highly atypical spindle cells with osteoid and adipose tissue. Fluorescence in situ hybridization (FISH) studies performed on the cell block demonstrated amplification of the *MDM2* gene. In addition, the findings were morphologically compatible with the previously resected retroperitoneal dedifferentiated liposarcoma with areas of osteosarcoma. This rare case illustrates the usefulness of FNA and ancillary studies in the diagnosis and subclassification of soft tissue tumors. To the best of our knowledge, this is the first report of *MDM2* FISH positivity in a liposarcoma diagnosed by FNA.

## INTRODUCTION

Liposarcoma (LPS) is a mesenchymal tumor that constitutes the most common type of soft tissue sarcoma in adults.[1,2] Of the subtypes of LPS, well-differentiated liposarcoma (WDLS) represents the largest group, accounting for about 40–50% of all cases. Dedifferentiation occurs in 10% of WDLS and the risk of dedifferentiation appears to be related to the location of the tumor, where it appears greatest in deep-seated tumors. Dedifferentiation in lipomatous tumors can occur in a primary or a recurrence, and involves a transition from a WDLS to a nonlipogenic sarcoma of variable histological grade.[1,2] In addition, dedifferentiated LPS has a less aggressive behavior when compared with other high-grade pleomorphic sarcomas, and thus, recognizing it is important from a clinical perspective.[3–5]

The murine double minute *(MDM2)* oncogene is important in controlling the cell cycle by binding to TP53 and promoting its degradation. For this reason, studies have looked at the role of *MDM2* in the pathogenesis of various tumors, including WDLS.[6] Detection of *MDM2* amplification has been done by immunohistochemistry or by gene amplification studies (PCR, comparative genomic hybridization/CGH, FISH), and has been shown to be useful in distinguishing WDLS and dedifferentiated LPS from benign mimics.[7–10]

We herein, describe a case of dedifferentiated LPS diagnosed by fine-needle aspiration (FNA) cytology and illustrate how fluorescent in-situ hybridization (FISH) studies can be helpful in confirming the diagnosis. In the cytology literature, there are only rare case reports of dedifferentiated LPS diagnosed on cytological material,[11] and to our knowledge, this is the first case report of a dedifferentiated LPS diagnosed by FNA with FISH studies performed on cytological material to confirm amplification of the *MDM2* oncogene.

## CASE REPORT

**Figure 1:**
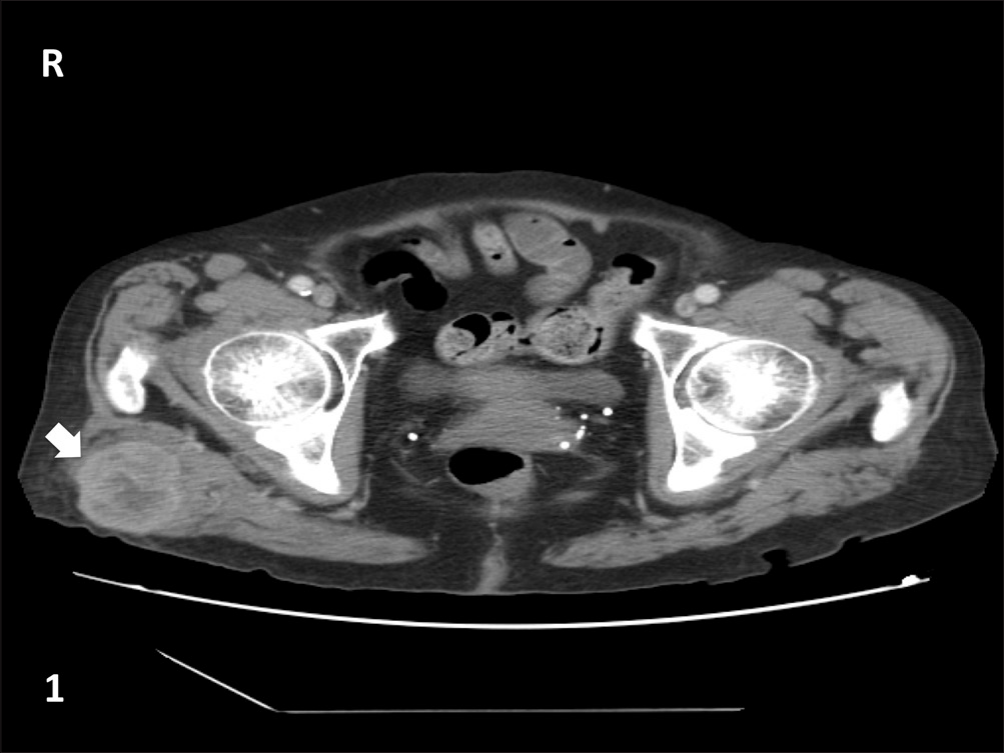
CT imaging of the pelvis with contrast. A contrast enhancing mass was identified in the right intra-gluteal muscle and measured 4.4 × 3.8 cm (arrow)

An 85-year old woman presented to our institution with a soft tissue mass in the right buttock, which was present for about four months. The mass had been increasing in size and was nontender. The patient's medical history included a retroperitoneal tumor excision 2 years prior to the onset of this lesion. On physical examination, the mass was firm, mobile, poorly circumscribed, and measured 4.4 × 3.8 cm. The CT scan with contrast, revealed a contrast-enhancing mass in the right gluteal soft tissue [Figure 1; arrow]. The patient was referred to the FNA clinic for an FNA biopsy.

The FNA biopsy was performed by a cytopathologist using palpation and 25-gauge needles. The aspirated material was used to make air-dried and alcohol-fixed smears, in addition to obtaining material in formalin for cell block preparation. The air-dried and alcohol-fixed smears were stained with Diff-Quik™ (Protocol Hema 3, Fisher Scientific, Kalamazoo, MI) and Papanicolaou technique, respectively. The on-site evaluation involved examination of the Diff-Quik™ stained smear from each FNA pass. A total of 5 passes were obtained, including one pass entirely for cell block.

At the time of final interpretation, the Papanicolaou and Diff-Quik™ stained smears, in addition to the hematoxylin and eosin (H and E) stained sections of the formalin-fixed cell block, were evaluated. For immunohistochemical stains, deparaffinized, formalin-fixed cell block sections were stained using a variety of different antibodies on the Ventana Benchmark XT system (Tucson, AZ). FISH studies for the *MDM2* amplification were requested on sections of the cell block on charged slides using probes for *MDM2*/CEP12 (MDM clones RP11-450G15 and RP11-775J10), which corresponds to locus 12q15/12p11.1q11.1 (CHORI, Oakland, CA/Vysis, Vysis, Abbott Laboratories, Abbott Park, IL). The *MDM2* FISH assay was scored by counting 65 nuclei under oil immersion at 100× magnification with a triple band pass filter. Only nuclei with at least two CEP12 signals were evaluated. The average number of *MDM2* and CEP12 signals was then determined and an *MDM2*/CEP12 ratio was calculated. A ratio of greater than or equal to 2.0 was considered amplified for the *MDM2* gene, whereas a ratio of less than 2.0 was considered nonamplified, as described in previous studies.[10]

### Cytomorphologic findings

**Figure 2:**
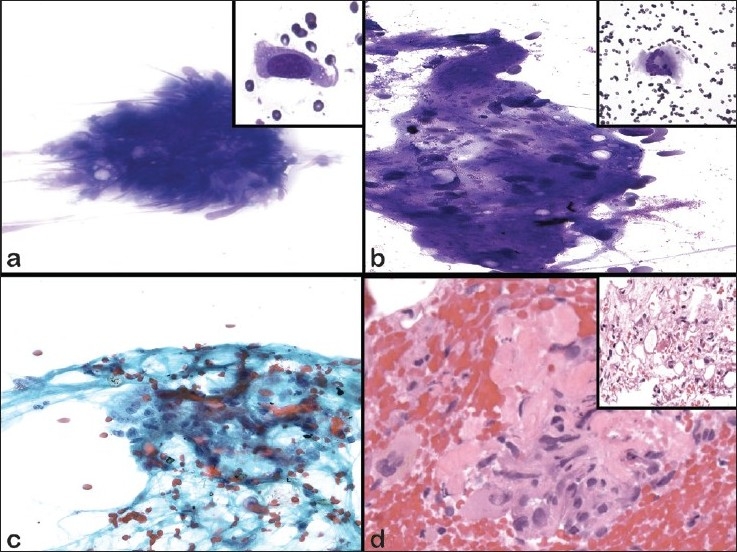
Cytomorphologic features of FNAB of dedifferentiated liposarcoma. a) DQ, ×400 (inset ×600); b) DQ, ×400 (inset ×200); c) Pap, ×400 (inset ×600); d) H&E, ×200). a) Loosely cohesive spindle cells with elongated atypical nuclei. Inset shows increased N/C ratio, hyperchromasia with tails of cytoplasm. b) Fragments of dense matrix with atypical spindle cells. Inset shows a multinucleated giant cell. c) Loosely cohesive spindle cells with enlarged hyperchromatic nuclei, cytoplasmic tails and coarse chromatin. d) Cell block sections shows cellular fragments of spindle cells with nuclear pleomorphism associated with dense eosinophilic matrix, consistent with osteoid. Inset shows areas of adipose tissue with scattered atypical cells

The aspirate smears revealed scattered cellular clusters [Figure 2a] and dyscohesive spindle cells [Figure 2A inset] with atypia in a background of adipose tissue and dense amorphous material [Figure 2b]. The spindle cells had enlarged, hyperchromatic oval nuclei with cytoplasmic tails and coarse chromatin [Figure 2a inset and 2c]. A few osteoclast-type giant cells were also noted [Figure 2b inset]. The cell block revealed hypercellular tissue fragments with pleomorphic spindle cells, including some with mitotic figures. The cells appeared to be intermixed with ribbons of dense eosinophilic material, which showed evidence of polarization and was consistent with osteoid [Figure 2d]. There was also an area of adipocytes associated with atypical stromal cells showing pleomorphism [Figure 2d inset]. No necrosis was identified.

The primary considerations in the differential diagnosis based on the cytomorphology was a spindle cell neoplasm, and included the possibility of a sarcomatoid carcinoma, spindle cell melanoma, pleomorphic sarcoma (otherwise referred to as malignant fibrous histiocytoma), and a recurrence/metastasis of the patient's previously resected soft tissue malignancy. Benign and reactive conditions were felt to be unlikely given the degree of cytologic atypia.

### Immunohistochemical findings

Immunohistochemical stains performed on sections of the cell block demonstrated that the tumor cells were positive for vimentin, focally positive for smooth muscle actin, and positive for MIB-1 (Ki-67) in 20% of the tumor nuclei. The tumor cells were negative for cytokeratin stains (AE1/AE3 and Cam 5.2), S-100, CD34, Melan-A, and HMB-45. The lack of cytokeratin staining, in addition to the lack of cohesive clusters, excluded the possibility of a sarcomatoid carcinoma. The negativity for S-100, MelanA, and HMB-45 immunohistochemical stains, in addition to the lack of prominent nucleoli and absence of melanin pigment, ruled out the possibility of a spindle cell melanoma. A malignant mesenchymal lesion was favored over a benign/reactive lesion due to the cytologic atypia present.

### FISH findings

**Figure 3:**
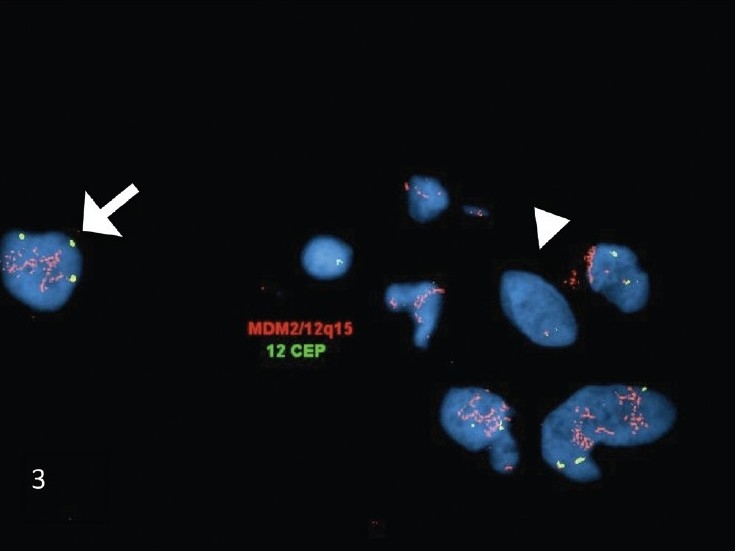
FISH studies performed on the FNA biopsy cell-block sections of dedifferentiated liposarcoma. The FISH studies demonstrated MDM2 (red signal) amplification in the atypical nuclei (arrow). In comparison, the adjacent normal cells showed no amplification of MDM2 (arrowhead). (The MDM2 probe is the red signal and the chromosome 12 centromere (CEP12) probe is the green signal)

FISH studies performed on sections of the cell block showed amplification of the *MDM2* gene, with a ratio of 27.5 [Figure 3]. The amplification of *MDM2* in the pleomorphic cells, further supported the cytomorphologic impression of a malignant spindle cell neoplasm, and essentially excluded the possibility of a benign/reactive spindle cell lesion.

### Histomorphologic findings

**Figure 4a-b:**
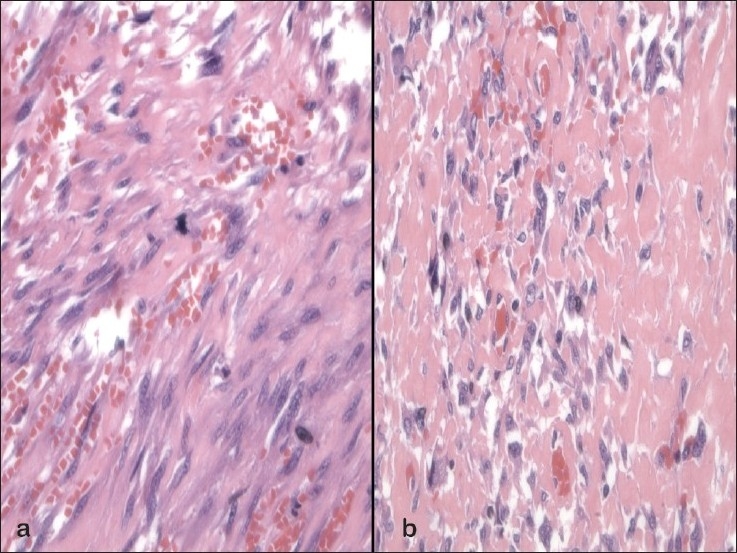
Histomorphologic features of dedifferentiated liposarcoma on excision. The excisional biopsy sections showed infiltrating malignant spindle cells with nuclear pleomorphism and mitoses. [a) Hematoxylin and eosin stain, ×600]. There were also areas of malignant spindle cells associated with ribbons of osteoid matrix [b) Hematoxylin and eosin stain, ×400]

The excision of the patient's previously diagnosed soft tissue tumor, which was located posterior to the kidney and within the psoas muscle, was reviewed with the FNA. It revealed a malignant spindle cell neoplasm with numerous mitotic figures, atypical stromal cells, and rare lipoblasts [Figure 4a]. In addition, there were areas of osteoid formation [Figure 4b], and other areas with sheets of osteoclast-type giant cells, xanthogranulomatous reaction, calcifications, and inflammation. The final diagnosis was dedifferentiated LPS with foci of well-differentiated LPS, in addition to areas of malignant osteosarcoma and areas resembling giant cell tumor.

## DISCUSSION

Our case demonstrates a case of dedifferentiated LPS that was diagnosed on FNA biopsy and was confirmed to have amplification of *MDM2* by FISH studies performed on the cell block. The presence of a heterologous component within a dedifferentiated LPS, such as seen in this case, is rare and only seen in about 10% of cases.[1,2] Furthermore, the admixture of different cell types in this FNA (scattered spindle cells, osteoclast-like giant cells, adipose tissue, and osteoid) made it difficult to determine if this was a benign/reactive process or a neoplastic process. This is a common dilemma in soft tissue FNA, where the cytologic distinction between benign spindle cell proliferations and spindle cell sarcomas is often difficult. For this reason, ancillary studies have been helpful in confirming and subclassifying certain soft tissue diagnoses.[12,13]

Of the available ancillary studies, FISH studies have been particularly helpful in soft tissue lesions, including lipomatous lesions. For example, characteristic chromosomal translocations involving chromosome 12q13 (*CHOP* gene) have been very advantageous in diagnosing myxoid LPS and differentiating it from other myxoid lesions.[14–17] Similarly, WDLS and dedifferentiated LPS have also been found to have characteristic chromosomal aberrations, such as ring or giant marker chromosomes composed of amplified targets within the 12q13-15 region,[18] which involve the *MDM2* and *CDK4* genes,[19] and can be detected by CGH, PCR, FISH, and/or immunohistochemical stains.[7–10] Of these techniques, IHC is very easy to perform, widely available, and easy to interpret. In addition, the antibodies have been shown to be relatively reliable in detecting overexpression of the protein from amplification of the corresponding gene.[7] The MDM2 immunostain appears to be more sensitive than the CDK4 immunostain for the detection of the *MDM2* amplification because CDK4 is not always co-amplified; however, the CDK4 immunostain is useful because it appears to be more specific. Thus, it is recommended to perform both immunostains together to maximize sensitivity and specificity. For interpretation of the IHC, a tumor is generally considered positive for MDM2 or CDK4 when at least one nucleus per high power field is positive.[7] However, of all the methods, it appears that FISH may be a more sensitive and specific method than PCR and IHC, in the limited studies comparing the methods.[8]

The detection of *MDM2* amplification has recently been shown to have diagnostic value in differentiating benign lipomatous lesions (including lipoma and pleomorphic/spindle cell lipoma) from WDLS and dedifferentiated LPS.[9,10] Approximately 95% of WDLS and dedifferentiated LPS showed *MDM2* gene amplification by FISH, and 97% of LPS show overexpression of MDM2 protein by immunohistochemistry.[7,8] In addition, *MDM2* gene amplification by FISH appears to be absent in benign lesions, making it a specific test with no false negatives in one study that looked at benign lesions and LPS.[9] The overall sensitivity and specificity for detection of WDLS and DLS were 97–100% and 83–92%, respectively by immunohistochemistry[7,9] and 88% and 100% by FISH studies.[9] However, studies looking at the value of *MDM2* amplification in the diagnosis of LPS have focused on excisional biopsies and resections, not cytological material. This case demonstrates that FISH for the *MDM2* amplification is a useful adjunct in diagnosing LPS in cytology; however, additional studies are needed to determine the sensitivity and specificity of this marker in cytological specimens.

In addition to LPSs, other sarcomas can show positivity for the *MDM2* immunostain and/or amplification of the *MDM2* gene, including atypical lipomatous tumors,[7] malignant peripheral nerve sheath tumors,[7,20] myxofibrosarcomas,[7] rhabdomyosarcomas,[7,21] and others.[7,22] In addition, other tumors aside from sarcomas can show *MDM2* amplification, including melanoma.[23] Thus, *MDM2* may be more helpful in deciding if a lesion is malignant, and may not be as helpful in specifically subtyping a neoplasm as a LPS. However, when looking at mesenchymal malignancies, it is uncertain whether the *MDM2* amplification truly occurs in a subset of pleomorphic sarcomas other than LPS, or whether this group simply represents dedifferentiated LPS that were under-sampled.[22,24] For example, one study looked at a series of malignant fibrous histiocytomas diagnosed in the retroperitoneum and most of these were reclassified as dedifferentiated LPS after careful analysis, including examination for *MDM2* and *CDK4* amplification by IHC and CGH.[25] In another study, Binh *et al*, calculated the sensitivity of MDM2 and CDK4 co-expression by immunohistochemistry for dedifferentiated LPS to be more than 90%, however, this immunostaining pattern is not absolutely specific for dedifferentiated LPS. They demonstrated this latter pattern of immunostaining in myxofibrosarcoma, leiomyosarcoma, MFH, MPNST, embryonal rhabdomyosarcoma and synovial sarcoma. Thus the immunohistochemical expression of MDM2 and CDK4 should be interpreted in view of the clinical and histomorphological context.[7]

This case also demonstrates some of the difficulties in soft tissue FNAs. From an interpretation standpoint, the diagnosis of sarcomas by FNA is challenging due to the cytomorphologic overlap in many soft tissue lesions, particularly in spindle cell lesions. From a technical perspective, soft tissue lesions with necrosis, dense fibrosis or matrix material (i.e. osteoid) can be challenging due to the scant cellularity of the aspirates. In these cases, concurrent core biopsies or dedicated FNA passes for cell block can help to obtain diagnostic material. In our case, the aspirates were only moderately cellular due to the firm nature of the lesion. However, dedicated passes for cell block showed small tissue fragments (“mini-biopsies”), which had osteoid and highly cellular groups of atypical spindle cells. Thus, we were able to acquire sufficient material to compare with the previously resected tumor and to perform ancillary studies, which was important in such a challenging case.

In conclusion, our case demonstrates the cytomorphological, histological, immunohistochemical, and cytogenetic findings in a case of recurrent dedifferentiated LPS with areas of osteosarcoma. To the best of our knowledge, the amplification of the *MDM2* oncogene and its usefulness in FNA biopsies of adipocytic soft tissue lesions has not been reported before in the cytology literature.

## CONCLUSION

This rare case of an 85-year old female with right gluteal soft tissue tumor illustrates the usefulness of FNA and ancillary studies in the diagnosis of a dedifferentiated LPS. Proper sampling of the lesion and obtaining sufficient material for the performance of ancillary studies (IHC and FISH) is critical. To our knowledge, this is the first report of *MDM2* amplification detected by FISH on cytologic material from an FNA.

## COMPETING INTEREST STATEMENT BY ALL AUTHORS:

No competing interest to declare by any of the authors.

## AUTHORSHIP STATEMENT BY ALL AUTHORS:

Each author acknowledges that this final version was read and approved. All authors of this article declare that we qualify for authorship as defined by ICMJE http://www.icmje.org/#author. Each author has participated sufficiently in the work and take public responsibility for appropriate portions of the content of this article.

## ETHICS STATEMENT BY ALL AUTHORS:

As this is case report without identifiers, our institution does not require approval from Institutional Review Board (IRB) (or its equivalent)
